# Association of Big Endothelin-1 with Coronary Artery Calcification

**DOI:** 10.1371/journal.pone.0142458

**Published:** 2015-11-13

**Authors:** Ping Qing, Xiao-Lin Li, Yan Zhang, Yi-Lin Li, Rui-Xia Xu, Yuan-Lin Guo, Sha Li, Na-Qiong Wu, Jian-Jun Li

**Affiliations:** 1 Division of Dyslipidemia, State Key Laboratory of Cardiovascular Disease, Fu Wai Hospital, National Center for Cardiovascular Diseases, Chinese Academy of Medical Sciences, Peking Union Medical College, Bei Li Shi Road 167, Beijing, 100037, China; 2 Department of Cardiology, Xingtai people's hospital, Red Star Street 16, Hebei, 054000, China; University of Sassari, ITALY

## Abstract

**Background:**

The coronary artery calcification (CAC) is clinically considered as one of the important predictors of atherosclerosis. Several studies have confirmed that endothelin-1(ET-1) plays an important role in the process of atherosclerosis formation. The aim of this study was to investigate whether big ET-1 is associated with CAC.

**Methods and Results:**

A total of 510 consecutively admitted patients from February 2011 to May 2012 in Fu Wai Hospital were analyzed. All patients had received coronary computed tomography angiography and then divided into two groups based on the results of coronary artery calcium score (CACS). The clinical characteristics including traditional and calcification-related risk factors were collected and plasma big ET-1 level was measured by ELISA. Patients with CAC had significantly elevated big ET-1 level compared with those without CAC (0.5±0.4 vs. 0.2±0.2, P<0.001). In the multivariate analysis, big ET-1 (Tertile 2, HR = 3.09, 95% CI 1.66–5.74, P <0.001, Tertile3 HR = 10.42, 95% CI 3.62–29.99, P<0.001) appeared as an independent predictive factor of the presence of CAC. There was a positive correlation of the big ET-1 level with CACS (r = 0.567, p<0.001). The 10-year Framingham risk (%) was higher in the group with CACS>0 and the highest tertile of big ET-1 (P<0.01). The area under the receiver operating characteristic curve for the big ET-1 level in predicting CAC was 0.83 (95% CI 0.79–0.87, p<0.001), with a sensitivity of 70.6% and specificity of 87.7%.

**Conclusions:**

The data firstly demonstrated that the plasma big ET-1 level was a valuable independent predictor for CAC in our study.

## Introduction

Coronary artery calcification (CAC) has long been known as an important part of atherosclerotic process. Previous autopsy investigations have found a significant association between the presence of CAC and atherosclerosis burden [1]. Now we can detect the quantification of CAC by electron-beam computed tomography (EBCT) [2]. In recent years, the evidence has tremendously increased that the presence of CAC can give prognostic information for subsequent coronary events in individuals with or without cardiovascular disease (CVD) [3].

Endothelin-1 (ET-1) is a pleiotropic molecule best known for its action as the most potent vasoconstrictor currently identified [4]. Previous studies have demonstrated increased ET-1 expression in atherosclerotic arteries compared with normal arteries in human [5]. However, circulating ET-1 has a very short half-life (40 to 70 s) [6]and it may be grossly underestimated.

Big ET-1, the precursor of ET-1, is a peptide of 38 amino acids, which is cleaved by ET converting enzyme-1 (ECE-1). It has been reported that plasma big ET-1 has longer half-life and easier to be detected. Moreover, emerging evidence suggested that big ET-1 is a more accurate indicator of the degree of activation of the endothelial system[7]. So it has been more widely used than ET-1 in most clinical researches.

The aim of this study, therefore, was to determine whether plasma big ET-1 was associated with CAC in patients who had a manifestation of chest pain.

## Subjects and Methods

### Study design and population

From Feb 2011 through May 2012, five hundred and ten consecutive outpatients who had a manifestation of chest pain and underwent cardiac CT using a 64-slice multidetector CT scanner were included in the study. All of them were referred to our hospital for test of blood, echocardiography and elective coronary angiography. All the blood samples within 24 hours and CAC scan within 1 month were taken. And the other imageological examinations were taken in 48 hours after withdrawing of blood samples. Mean age of patients was 56 ±10 years and 350 of them were males.

Coronary arterial disease (CAD) was defined as ≥50% luminal diameter stenosis of at least one major epicardial coronary artery in elective coronary angiography. Hypertension was defined as a repeated blood pressure of ≥140/90 mmHg (at least two times in different environments) or the use of antihypertensive drugs. Diabetes mellitus (DM) was defined as a fasting serum glucose level of ≥6.99 mmol/L on multiple occasions or the use of treatment with insulin or oral hypoglycemic agents. Patients with a history of heart failure or cardiomyopathies, renal dysfunction, hepatic failure, hemolytic disorders, concomitant inflammatory diseases, neoplastic diseases, thyroid disease, acute infectious/inflammatory conditions, and a history of coronary revascularization (percutaneous coronary intervention or coronary artery bypass graft surgery) were excluded from the study. Cardiovascular risk was assessed by Framingham risk score [8].

The study complied with the Declaration of Helsinki, and was approved by the hospital ethics review board (Fu Wai Hospital & National Center for Cardiovascular Diseases, Beijing, China). Informed written consent was obtained from all patients included in this analysis.

### Laboratory examination

Venous blood samples were obtained from each patient at baseline upon admission. Each blood sample was immediately placed on ice and then centrifuged for 20 minutes at 4°C. Plasma separation was subsequently performed at −4°C, and the samples would be tested in two hours after blood taking. Total cholesterol (TC) was measured by enzymatic methods and the levels of Hemoglobin A1c (HbA1c) were measured using the Tosoh G7 Automate HPLC Analyzer (TOSOH Bioscience, Japan). The plasma big ET-1 level was measured using a highly sensitive and specific commercial sandwich enzyme immunoassay (BI-20082H, Biomedica, Wien, Austria). The levels of high sensitivity-CRP (hs-CRP) were determined using immunoturbidimetry (BeckmannAssay360, Bera,Calif.,USA).

All other included biomarkers were analyzed by standard hematological and biochemical tests. The patients were classified into 3 tertiles according to their plasma big ET-1 level: Tertile 1 included patients with plasma big ET-1 between 0.08 to 0.23 pmol/L, Tertile 2: 0.24 to 0.52 pmol/L, Tertile 3: 0.53 to 3.70 pmol/L.

### Coronary angiography and Echocardiography

Elective coronary angiography was performed for all enrolled patients using the standard Judkins technique, and the results were analyzed by at least two interventional physicians, as in our previous study[9]. Only angiograms with visually smooth contours with no wall irregularities were considered to be normal. The patients underwent echocardiography and ventriculography for measuring the left ventricular ejection fraction(LVEF).

### Measurement of CACS

All computerized tomography scans were performed on a 64-row spiral CT scanner (Light Speed VCT, GE Healthcare, Milwaukee, Wisconsin) with a 0.35-second rotation time with a pitch of 0.16 to 0.22, tube voltage of 120 kV, and tube current of 200 to 550 mA. Patients with heart rates >70 beats/min were given 25 mg to 50 mg of metoprolol (Selokeen, AstraZeneca, Zoetermeer, the Netherlands) orally 1 h before scanning, unless they had known any contraindication for beta-blocker usage. All data sets were reconstructed using retrospective electrocardiography (ECG)-gated sequential scan during 40% to 80% RR interval. Data sets were used to a dedicated workstation (Deep Blue, ADW4.3, GE Healthcare). Calcium was defined as the presence of at least 3 contiguous pixels with a density 130 HU. The calcium score for each artery was the sum of calcium scores of the left main, left anterior descending, left circumflex and right coronary arteries according to the Agatston scoring algorithm [10].

### Statistical analysis

All analyses were performed using the SPSS version 19.0. software program (Chicago, Illinois, USA). Continuous variables were expressed as means±standard deviation and categorical variables were expressed as frequencies with percentages. All continuous variables were checked with Kolmogorov–Smirnov normality test to show their distributions. Continuous variables with normal distributions were compared using the two-sample Student’s t-test and analysis of variance (ANOVA). Continuous variables with abnormal distributions were compared using the Kruskall wallis test. For categorical variables, the Chi-square test was used.

The patients were grouped according to their plasma big ET-1 levels in three tertiles. We compared the risk factors for CAD and CAC between the big ET-1 tertiles. The relationship between big ET-1 and CACS, age, male gender, DM, hypertension, smoking status, serum creatinine, uric acid, TC, HbA1c, hs-CRP and LVEF were examined by Pearson's correlation analyses. A multivariable logistic regression analyses were performed to identify variables with a significant independent association with absence of CAC. To assess the value of the Big ET-1 as a predictor for CAC, receiver-operator characteristic (ROC) curve was used. To determine the locally appropriate cut-off point for the Big ET-1, the Youden index (sensitivity+specificity−1) was calculated and the corresponding cut-off value for the highest Youden index was considered as the optimal cut-off value. Post-hoc analysis using the Bonferroni analysis was done to evaluate the p values for each pair of comparison (in the figures).

The alternative test hypothesis was built as two-sided for each statistical analysis. The tests were independent and so the experimentwise Type I error does not exceed 0.05 alpha levels. Significant univariate variables with P<0.05 were included in the multiple logistic regression analysis for odds ratios and 95% confidence intervals. A p-value of less than 0.05 was considered to be statistically significant.

## Results

### Baseline clinical characteristics

**Fig 1:**
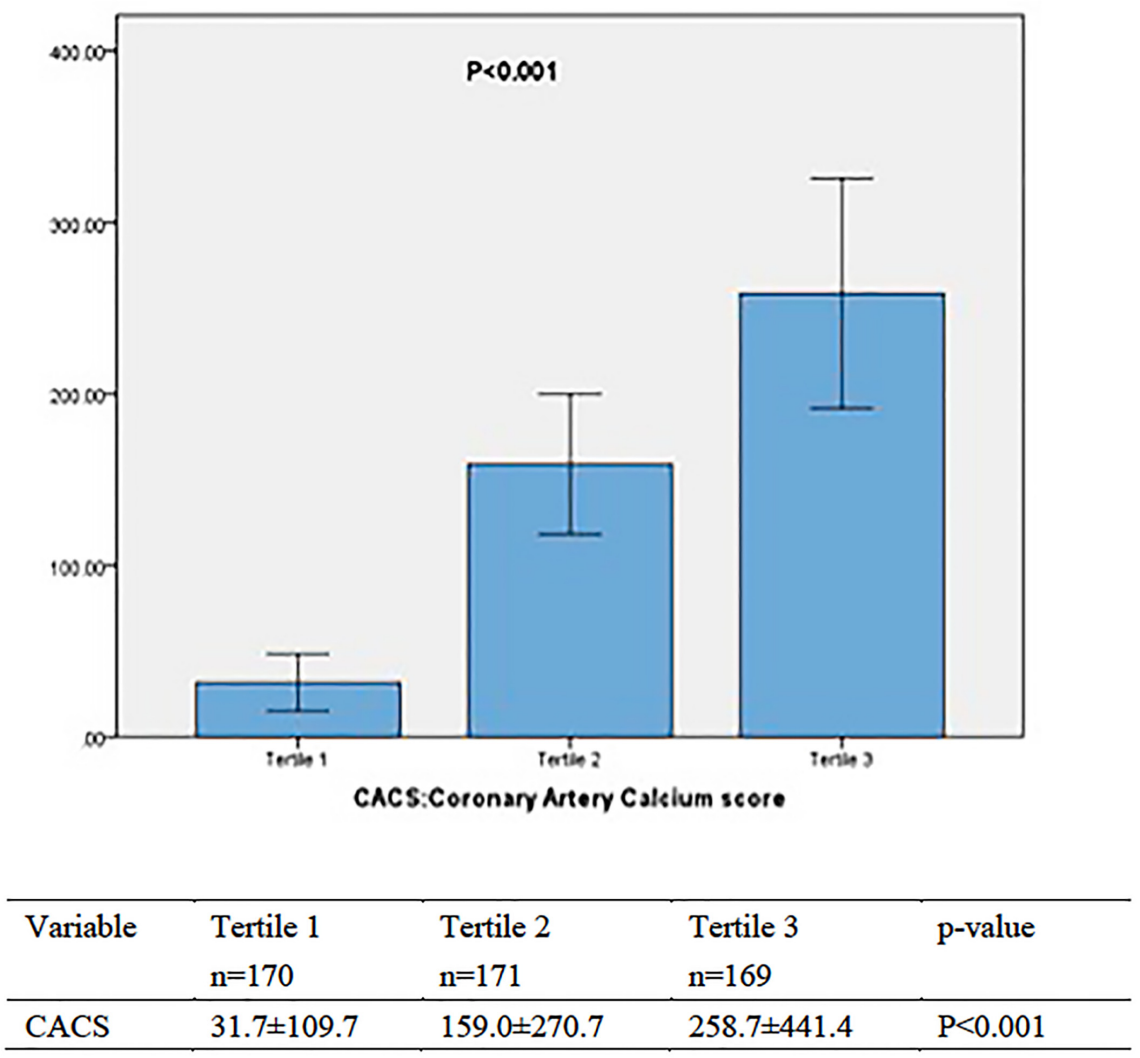
Relation between Coronary Artery Calcium score and Big ET-1 tertiles. Tertile 1: Patients with Plasma Big ET-1 level between 0.08 to 0.23 pmol/L; Tertile 2: Patients with Plasma Big ET-1 level between 0.24 to 0.52 pmol/L; Tertile 3: Patients with Plasma Big ET-1 level between 0.53 to 3.70 pmol/L.

Baseline clinical and laboratory characteristics of patients according to big ET-1 tertiles were shown in Table 1. Participants in higher big ET-1 tertile had elevated CACS (P<0.001, Fig 1). Patients in Tertile 1 (big ET-1 0.08 to 0.23 pmol/L) had a mean CACS of 31.7±109.7 (range:0–926.0, median:0). Patients in Tertile 2 (big ET-1 0.24 to 0.52 pmol/L) had a mean CACS of 159.0±270.7 (range:0–1376.0, median:26.3). Patients in Tertile 3 (big ET-1 0.53–3.70 pmol/L) had a mean CACS of 258.7±441.4 (range:0–3391.0, median:105.0).

**Table 1:** Baseline characteristics in patients with unadjusted risk factor levels according to plasma Big ET-1 concentration tertiles.

		Plasma Big ET-1 tertiles			
	All patients	Tertile 1	Tertile 2	Tertile 3	P value
		0.08–0.23	0.24–0.52	0.53–3.70	
	n = 510	n = 170	n = 171	n = 169	
Age (year)	56±10	51±10	57±10	60±10	<0.001
Gender, male, n (%)	350(68.6)	118(23.1)	110(21.6)	122(23.9)	0.285
BMI(kg/m^2^)	25.7±3.1	25.9±3.0	25.7±3.2	25.6±3.1	0.66
CAD, n(%)	268(52.5)	32(18.8)	90(52.6)	146(86.4)	<0.001
Diabetes mellitus, n (%)	120(23.5)	22(4.3)	38(7.5)	60(11.8)	<0.001
Hypertension, n (%)	304(59.6)	84(16.5)	103(20.2)	117(22.9)	0.001
Smoking, n (%)	232(45.5)	61(12.0)	72(14.1)	99(19.4)	<0.001
ACEI or ARB, n (%)	63(12.4)	7(1.4)	21(4.1)	35 (6.9)	<0.001
Statin, n (%)	152(29.8)	27(5.3)	56(11.0)	69(13.5)	<0.001
Creatinine (mmol/l)	76.2±15.0	77.7±15.3	73.9±14.6	77.0±14.9	0.045
TC (mmol/L)	4.7±1.0	4.9±0.9	4.7±1.1	4.4±1.0	<0.001
HbA1c (%)	6.2±1.0	5.9±0.9	6.2±0.8	6.4±1.2	<0.001
hs-CRP (mg/L)	2.5±3.0	2.2±2.7	2.2±2.7	3.1±3.4	0.003
CACS	149.7±318.5	31.7±109.7	159.0±270.7	258.7±441.4	<0.001
LVEF (%)	64.2±6.9	65.4±4.9	63.9±6.8	63.41±8.5	0.024

Participants in higher big ET-1 tertiles were older, more likely to be smoking and associated with CAC, DM and hypertension. While they had elevated HbA1c, hs-CRP levels and low TC levels (Table 1; P<0.05 for tertile trend).

### Big ET-1 and CAC

Positive correlation was found between big ET-1 and CACS (r = 0.567,P<0.001), age (r = 0.284,P<0.001), smoking status (r = 0.184,P<0.001), CAD (r = 0.387,P<0.001), DM (r = 0.142,P = 0.001), hypertension (r = 0.100,P = 0.025), HbA1c (r = 0.111,P<0.001) and hs-CRP (r = 0.138, P = 0.002, Table 2). Negative correlation was found between big ET-1 and LVEF(r = -0.164, P<0.001, Table 2). Comparison within the groups by Bonferroni post hoc analysis showed participants in higher big ET-1 tertiles had elevated CACS (P<0.001, Fig 1; Tertile 1 vs. Tertile 2, Tertile 1 vs. Tertile 3, Tertile 2 vs. Tertile 3, all P<0.001, S1 Table). Patients were stratified into the two groups according to the presence of CAC: CACS = 0 and CACS>0 for the purpose of examining whether big ET-1 is an independent predictor of CAC. In the univariate analysis (Table 3), age, gender, CAD, DM, hypertension, smoking, TC, HbA1c and big ET-1 were significantly correlated with the presence of CAC.

**Table 2:** Correlation between of Big ET-1 and other variables.

	All patients	
	r	P
CACS	0.567	<0.001
Age	0.284	<0.001
Gender, male	-0.003	0.945
BMI	-0.036	0.416
CAD	0.387	<0.001
Diabetes mellitus	0.142	0.001
Hypertension	0.100	0.025
Smoking	0.184	<0.001
ACEI or ARB	0.210	<0.001
Statin	0.217	<0.001
Creatinine	-0.006	0.900
Total cholesterol(mmol/L)	-0.032	0.678
HbA1c(%)	0.111	<0.001
hs-CRP(mg/L)	0.138	0.002
LVEF(%)	-0.164	<0.001

**Table 3:** Univariate analysis for presence of coronary artery calcium.

	Patients without CAC	Patients with CAC	P value
	N: 146	N: 364	
Age(year)	49±9	59±10	<0.001
Gender, male, n(%)	89(61.0)	261(71.7)	0.020
BMI(kg/m^2^)	25.8±3.0	25.7±3.1	0.687
CAD, n(%)	8(5.5)	260(71.4)	<0.001
Diabetes mellitus,n(%)	15(10.3)	105(28.8)	<0.001
Hypertension, n(%)	66(45.2)	238(65.4)	<0.001
Smoking, n(%)	49(33.6)	183(50.3)	0.001
ACEI or ARB	3(2.1)	60(16.5)	<0.001
Statin	35(24.0)	117(32.1)	0.070
Creatinine(mmol/l)	75.4±13.8	76.5±15.5	0.459
Total cholesterol(mmol/L)	4.9±1.0	4.6±1.0	0.005
HbA1c(%)	5.8±0.8	6.2±1.1	<0.001
hs-CRP(mg/L)	2.2±2.4	2.6±3.1	0.374
Big ET-1(pmol/L)	0.2±0.2	0.5±0.4	<0.001
LVEF(%)	65.0±5.9	64.0±6.6	0.095

Controlled for major potential confounders which p-values are less than 0.05 in the univariate analysis (including age, gender, smoking, CAD, hypertension, TC, HbA1c, hs-CRP) and drugs which may affect plasma big ET-1 level (such as ACEI, ARB and statin), the tertiles of big ET-1 were analysised in the multivariate analysis by Cox regression model (Table 4). The final data suggested that, apart from several factors, big ET-1 was remained as an independent predictor for the patients with CAC (Tertile 2, HR = 3.09, 95% CI 1.66–5.74, P <0.001, Tertile 3 HR = 10.42, 95% CI 3.62–29.99, P<0.001).

**Table 4:** Multivariate analysis for presence of coronary artery calcium.

Variable	OR	95% CI	P value
Age	1.11	1.07–1.16	<0.001
Gender	5.08	2.33–11.09	<0.001
CAD	22.29	9.19–54.04	<0.001
Hypertension	0.66	0.37–1.18	0.158
smoking	1.48	0.75–2.94	0.262
ACEI or ARB	3.44	0.72–16.52	0.122
Statin	0.22	0.09–0.53	0.001
Total cholesterol	0.86	0.63–1.17	0.331
HbA1c	1.10	0.75–1.63	0.623
Big ET-1,Tertile 1	1.00	-	-
Big ET-1,Tertile 2	3.09	1.66–5.74	<0.001
Big ET-1,Tertile 3	10.42	3.62–29.99	<0.001

**Fig 2:**
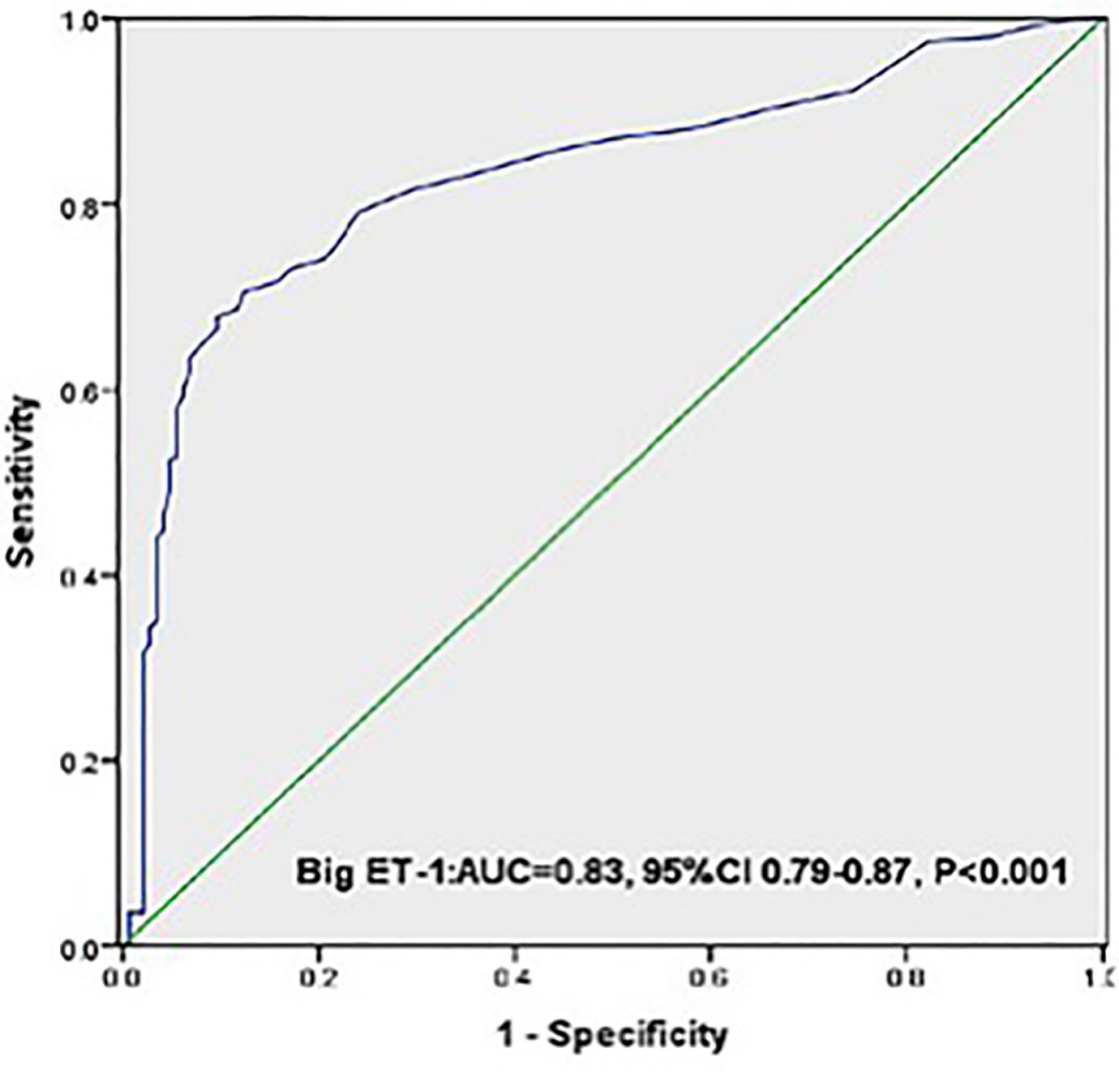
ROC curves for the Big ET-1 and HbA1c. ROC curves showed discriminatory power of baseline Big ET-1 and HbA1c on population with CAC.

Area under the receivers operating characteristic (ROC) curves (Fig 2 and Table 4) suggested that big ET-1, beyond lipid parameters and nonspecific inflammatory biomarkers, was a significant predictor for patients with CAC (AUC = 0.83, 95% CI 0.79–0.87, P<0.001). The optimal cutoff value for the plasma big ET-1 level for predicting CAC was 0.30 pmol/L, with a sensitivity of 70.6% and specificity of 87.7%.

### 10-Year Framingham Risk

**Fig 3:**
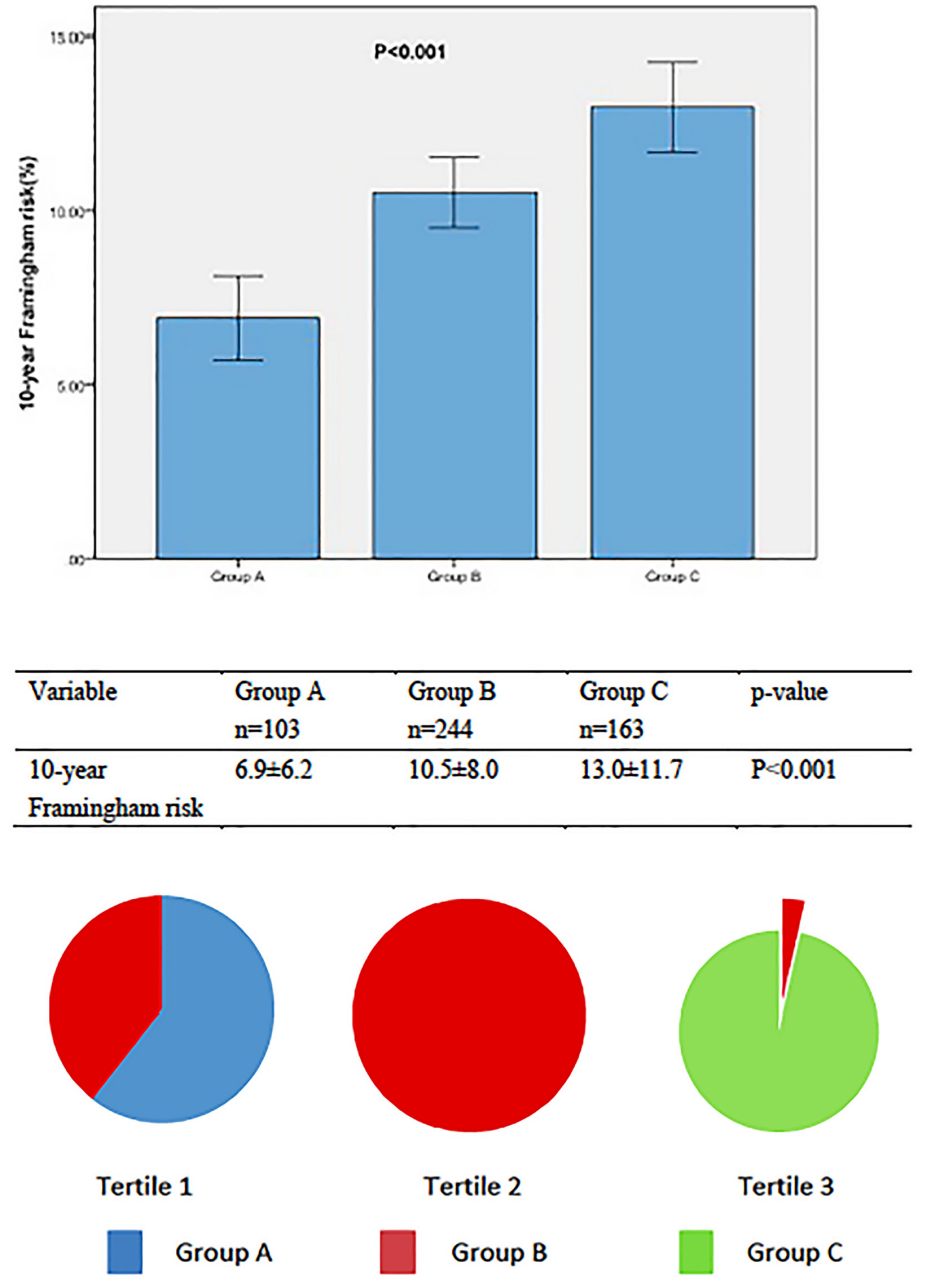
The 10-year Framingham risk in 3 subgroups of patients. The risk is lower in individuals with both CACS = 0 and Tertile 1 of Big ET-1. Group A: CACS = 0 and Tertile 1 of Big ET-1; Group C: CACS >0 and Tertile 3 of Big ET-1; Group B: Others.

To evaluate the value of the 10-year Framingham risk using combination of big ET-1 and CAC in our study, patients were divided into three subgroups according to plasma big ET-1 levels and CAC. Group A (n = 103): CACS = 0 and the levels of big ET-1 in Tertile 1; Group C (n = 163): CACS>0 and the levels of big ET-1 in Tertile 3; Group B (n = 244): the participants who were not in Group A and in Group C. Comparison within the groups by Bonferroni post hoc analysis showed the combintion of big ET-1 and CAC was a significant predictor for the 10-year Framingham risk. The 10-year Framingham risk (%) was 6.9±6.2 in group A, 10.5±8.0 in group B and 13.0±11.7 in group C (P<0.001, Fig 3; Group A vs. B, Group A vs. C, Group B vs. Group C, all P<0.001, S2 Table).

## Discussion

The present study is the first clinical investigation to demonstrate the correlation between big ET-1 and CAC in patients without renal dysfunction. The data for the first time showed that plasma big ET-1 level was an independent risk factor for presence of CAC, and that Big ET-1 had a linear relationship with CACS. In addition, the Framingham risk scores were significantly lower in the individuals with both absence of CAC and lower levels of big ET-1 than that in the other groups which CACS>0 or had higher levels of big ET-1. Additionally, similar to previous studies, the results of our study indicated that age, sex and CAD were as independent predictive factors of the presence of CAC.

Vascular calcification, which was considered as a passive degenerative condition of aging previously, is currently characterized as an active biomineralization process, more akin to bone formation [11]. Pathologically, it is the deposition of apatite mineral in the intimal or medial layers of the vessel wall and is clinically seen in atherosclerosis, diabetes, chronic kidney disease and aging. In fact, there are two main types of vascular calcification have been reported: intimal calcification and medial arterial calcification. The former is related to coronary atherosclerosis, whereas the latter is not associated with lipid deposition or inflammation but metabolic disorders, such as uremia and DM. As we well known, CAC has long been known as an important part of atherosclerotic process and it occurs almost all stages of the atherosclerosis of coronary in autopsy investigations[12]. The progression of calcification may begin with microcalcification (≥0.5 μm), which is seen in the early stage of atherosclerosis [13]. According to the literature, CT can only identify calcification areas of 1.03 to 1.37 mm in size [14]. Therefore, microcalcification will not be identified by CT. Only once aggregation of calcium occurs in fibroatheromas can be identified by CT scan[15].

In recent years, the evidence has tremendously suggested that the presence of CAC can give prognostic information for subsequent coronary events in individuals with or without CVD [3]. In the 2010 ACCF/AHA guideline for assessment of cardiovascular risk in asymptomatic adults, CAC was recommended for risk stratifying individuals at intermediate risk [16]. The Multi-Ethnic Study of Atherosclerosis (MESA) even showed that the CAC performed better than traditional risk factors as a predictor of events [17,18].

Although CAC has been regarded as a predictor of CV events, it remains unclear whether CAC is a benign or ominous finding. The clinical imaging studies conducted in patients with acute coronary syndrome have shown a higher proportion of non-calcified plaque compared with calcified plaque[19,20]. Serial studies have shown that severe calcified plaques are more resistant to change in atheroma burden compared with less calcified plaques[21]. Megan Coylewright et al. found that those with very high CACS (ACS high e with NREF_21" \have acute ischemic events compared to less severe disease (400–999)[22]. These findings support the concept that CAC is a part of healing process and confer stability to atherosclerotic plaques rather than the opposite.

ET-1, a peptide of 21 amino acid residues, is one of a family of three proteins encoded by distinct genes that also includes Endothelin-2 (ET-2) and Endothelin-3 (ET-3). ET-1 is regarded as one of the most potent endogenous vasoconstrictor so far[4]. It is the predominant isoform produced by endothelial cells and also expressed in vascular smooth muscle cell (VSMC), cardiac myocytes, macrophages, various neurons and other cells [23]. But ET-1 is unstable because it may be regulated by many factors such as Angiotensin II, nitric oxide, prostacyclin, shear stress, and drugs [24–26]. It has been reported that statin could significantly decrease the level of ET-1 in circulation [27]. Extensive studies indicate that ET-1 is one of the important pathogeneses of atherosclerosis. Increased expression of ET-1 has been found in atherosclerotic animal models [28]as well as in human atherosclerotic coronary artery[29]. It is bound to result in enhanced vasoconstrictor tone, increased inflammatory activity and elevated oxidative stress.

Since ET-1 and CAC were closely related to atherosclerosis respectively, the relationship between them is need of being confirmed. Although elevated ET-1 prompts endothelial injury or early atherosclerosis [30], it has not been regarded as a marker because of its unsustainability. CAC is considered as a part of the healing mechanism of atherosclerotic process. Whereas it only can be detected in the late stages of atherosclerosis due to the limitation of examination by CT scan [15]. Both of them have been shown to be highly predictive for the future CV events [18,31]. However, because of the nature of a special or limited mean of detection, alone as a predictive indicator remains controversial. Our data showed combination of big ET-1 and CAC had unexpected good predictive value.

Although there are few studies regarding the relationship between ET-1 and CAC, the positive correlation of ET-1 with the aorta or mitral valve calcification has already been confirmed. Wu SY et al. demonstrated endothelin content and mRNA were significantly increased in calcified VSMCs and calcified aorta in vivo[32]. In a histologic as well as histochemical study, Tuomas et al. found expression in human calcified aortic valves, the tissue levels of biologically active ET-1 was much higher than that of the normal ones [33]. Some studies have investigated the relationship between endothelial function and CAC. In the retrospective study, JING et al. demonstrated that the absence of CAC has a strong correlation with normal endothelial function in patients with non-cardiovascular disease and non-diabetes [34]. While Huang et al. reported that increased CAC strongly predicted endothelial dysfunction in patients with suspected CAD [35].

Although some studies have displayed the association of ET-1 or endothelial function with calcification, the underlying mechanisms of these relationships remain unclear. There are some proposed mechanisms. Firstly, ET-1 may cause artery calcification through its potential to promote VSMC phenotypes changed [36]. Essalihi R1 and his research group showed that an endothelin receptor antagonist could regress medial arterial calcification in a rat model produced by chronic warfarin treatment as early as in 2002 [37]. Moreover, ET-1 may accelerate artery calcification due to its ability of inducing VSMC apoptosis. The shear stress may upregulate the secretion of ET-1 and the increased arterial loading is further accentuated by the presence of ET-1, which will lead to VSMC apoptosis [38]. Finally, ET-1 may reduce the inhibitor of vascular calcification. Moe SM, et al. found the inverse relationship between serum fetuin-A and ET-1 coupled with the negative correlation of CACS with fetuin-A levels [39]. The study from Mustonen E, et al showed that ET-1 could decrease matrix Gla protein mRNA levels by 30% in myocytes[40].

There are several limitations in the present study. First, most of the study patients were admitted to assess their CAD risk and had symptoms of chest pain at the time of CT scanning. Therefore, our patient population was different from the general population to implicate our finding to general population. Moreover, our study did not permit the determination of causality due to a cross-sectional feature. Moreover, a single center source of patients may also be a limitation. Finally, the results from relative small sample size suggested that a large cohort study is needed.

## Conclusions

In conclusion, the present data demonstrated that the plasma big ET-1 level was a valuable independent predictor of CAC in our study. The combination of big ET-1 and CAC is in good accordance with the Framingham risk score.

## Supporting Information

S1 Table Comparison the level of CACS between ET-1 tertiles. (DOCX)

S2 Table The10-year Framingham risk between groups. (DOCX)

## Abbreviations

ET-1: endothelin-1
CAC: coronary artery calcification
CACS: coronary artery calcium score
CAD: coronary artery disease
CVD: cardiovascular disease
MESA: Multi-Ethnic Study of Atherosclerosis
BMI: body mass index
ACEI: angiotensin converting enzyme inhibitor
ARB: angiotensin receptor inhibitor
HDL: high-density lipoprotein
LDL: low-density lipoprotein
HbA1c: glycosylated hemoglobin A1C
hs-CRP: high-sensitivity C-reactive protein
LVEF: left ventricular ejection fraction
VSMC: vascular smooth muscle cell

## References

[1] Sangiorgi G, Rumberger JA, Severson A, Edwards WD, Gregoire J, et al (1998) Arterial calcification and not lumen stenosis is highly correlated with atherosclerotic plaque burden in humans: a histologic study of 723 coronary artery segments using nondecalcifying methodology. J Am Coll Cardiol 31: 126–133. 9426030 10.1016/s0735-1097(97)00443-9

[2] Wexler L, Brundage B, Crouse J, Detrano R, Fuster V, et al (1996) Coronary artery calcification: pathophysiology, epidemiology, imaging methods, and clinical implications. A statement for health professionals from the American Heart Association. Writing Group. Circulation 94: 1175–1192. 8790070 10.1161/01.cir.94.5.1175

[3] Yamamoto H, Ohashi N, Ishibashi K, Utsunomiya H, Kunita E, et al (2011) Coronary calcium score as a predictor for coronary artery disease and cardiac events in Japanese high-risk patients. Circ J 75: 2424–2431. 21778594 10.1253/circj.cj-11-0087

[4] Kawanabe Y, Nauli SM (2011) Endothelin. Cell Mol Life Sci 68: 195–203. 10.1007/s00018-010-0518-0 20848158 PMC3141212

[5] Ihling C, Szombathy T, Bohrmann B, Brockhaus M, Schaefer HE, et al (2001) Coexpression of endothelin-converting enzyme-1 and endothelin-1 in different stages of human atherosclerosis. Circulation 104: 864–869. 11514370 10.1161/hc3301.094742

[6] Jordan W, Decker M, Kamrowski H, Brunner E, Ehrenreich H, et al (2002) Effects of cerebrovascular challenges on plasma endothelin. Neurosci Res 43: 127–134. 12067748 10.1016/s0168-0102(02)00022-6

[7] Kolettis TM (2014) Ventricular tachyarrhythmias during acute myocardial infarction: The role of endothelin-1. Life Sci. 10.1016/j.lfs.2014.01.060 24486303

[8] Wilson PW, D'Agostino RB, Levy D, Belanger AM, Silbershatz H, et al (1998) Prediction of coronary heart disease using risk factor categories. Circulation 97: 1837–1847. 9603539 10.1161/01.cir.97.18.1837

[9] Li JJ, Nie SP, Qian XW, Zeng HS, Zhang CY (2009) Chronic inflammatory status in patients with coronary artery ectasia. Cytokine 46: 61–64. 10.1016/j.cyto.2008.12.012 19232498

[10] Agatston AS, Janowitz WR, Hildner FJ, Zusmer NR, Viamonte M Jr., et al (1990) Quantification of coronary artery calcium using ultrafast computed tomography. J Am Coll Cardiol 15: 827–832. 2407762 10.1016/0735-1097(90)90282-t

[11] Sage AP, Tintut Y, Demer LL (2010) Regulatory mechanisms in vascular calcification. Nat Rev Cardiol 7: 528–536. 10.1038/nrcardio.2010.115 20664518 PMC3014092

[12] Mautner GC, Mautner SL, Froehlich J, Feuerstein IM, Proschan MA, et al (1994) Coronary artery calcification: assessment with electron beam CT and histomorphometric correlation. Radiology 192: 619–623. 8058924 10.1148/radiology.192.3.8058924

[13] Kelly-Arnold A, Maldonado N, Laudier D, Aikawa E, Cardoso L, et al (2013) Revised microcalcification hypothesis for fibrous cap rupture in human coronary arteries. Proc Natl Acad Sci U S A 110: 10741–10746. 10.1073/pnas.1308814110 23733926 PMC3696743

[14] Lusis AJ (2000) Atherosclerosis. Nature 407: 233–241. 11001066 10.1038/35025203 PMC2826222

[15] Rumberger JA, Simons DB, Fitzpatrick LA, Sheedy PF, Schwartz RS (1995) Coronary artery calcium area by electron-beam computed tomography and coronary atherosclerotic plaque area. A histopathologic correlative study. Circulation 92: 2157–2162. 7554196 10.1161/01.cir.92.8.2157

[16] Greenland P, Alpert JS, Beller GA, Benjamin EJ, Budoff MJ, et al (2010) 2010 ACCF/AHA guideline for assessment of cardiovascular risk in asymptomatic adults: a report of the American College of Cardiology Foundation/American Heart Association Task Force on Practice Guidelines. J Am Coll Cardiol 56: e50–103. 10.1016/j.jacc.2010.09.001 21144964

[17] Budoff MJ, Nasir K, McClelland RL, Detrano R, Wong N, et al (2009) Coronary calcium predicts events better with absolute calcium scores than age-sex-race/ethnicity percentiles: MESA (Multi-Ethnic Study of Atherosclerosis). J Am Coll Cardiol 53: 345–352. 10.1016/j.jacc.2008.07.072 19161884 PMC2652569

[18] Martin SS, Blaha MJ, Blankstein R, Agatston A, Rivera JJ, et al (2014) Dyslipidemia, coronary artery calcium, and incident atherosclerotic cardiovascular disease: implications for statin therapy from the multi-ethnic study of atherosclerosis. Circulation 129: 77–86. 10.1161/CIRCULATIONAHA.113.003625 24141324 PMC3919521

[19] Schuijf JD, van der Wall EE, Bax JJ (2009) Lesions without calcium: lessons from CT angiography. Heart 95: 1038–1040. 10.1136/hrt.2008.164582 19389721

[20] Otsuka F, Finn AV, Virmani R (2013) Do vulnerable and ruptured plaques hide in heavily calcified arteries? Atherosclerosis 229: 34–37. 10.1016/j.atherosclerosis.2012.12.032 23375681

[21] Nicholls SJ, Tuzcu EM, Wolski K, Sipahi I, Schoenhagen P, et al (2007) Coronary artery calcification and changes in atheroma burden in response to established medical therapies. J Am Coll Cardiol 49: 263–270. 17222740 10.1016/j.jacc.2006.10.038

[22] Coylewright M, Rice K, Budoff MJ, Blumenthal RS, Greenland P, et al (2011) Differentiation of severe coronary artery calcification in the Multi-Ethnic Study of Atherosclerosis. Atherosclerosis 219: 616–622. 10.1016/j.atherosclerosis.2011.08.038 21930271

[23] Barton M (2014) Aging and endothelin: Determinants of disease. Life Sci. 10.1016/j.lfs.2014.09.009 25239727

[24] Lin YJ, Kwok CF, Juan CC, Hsu YP, Shih KC, et al (2014) Angiotensin II enhances endothelin-1-induced vasoconstriction through upregulating endothelin type A receptor. Biochem Biophys Res Commun 451: 263–269. 10.1016/j.bbrc.2014.07.119 25088996

[25] Rapoport RM (2014) Nitric Oxide Inhibition of Endothelin-1 Release in the Vasculature: In Vivo Relevance of In Vitro Findings. Hypertension. 10.1161/HYPERTENSIONAHA.114.03837 25135184

[26] Ishibazawa A, Nagaoka T, Takahashi T, Yamamoto K, Kamiya A, et al (2011) Effects of shear stress on the gene expressions of endothelial nitric oxide synthase, endothelin-1, and thrombomodulin in human retinal microvascular endothelial cells. Invest Ophthalmol Vis Sci 52: 8496–8504. 10.1167/iovs.11-7686 21896842

[27] Zhang X, Li Q, Zhao J, Li X, Sun X, et al (2014) Effects of combination of statin and calcium channel blocker in patients with cardiac syndrome X. Coron Artery Dis 25: 40–44. 10.1097/MCA.0000000000000054 24256699

[28] Barton M, Haudenschild CC, d'Uscio LV, Shaw S, Munter K, et al (1998) Endothelin ETA receptor blockade restores NO-mediated endothelial function and inhibits atherosclerosis in apolipoprotein E-deficient mice. Proc Natl Acad Sci U S A 95: 14367–14372. 9826706 10.1073/pnas.95.24.14367 PMC24379

[29] Zeiher AM, Ihling C, Pistorius K, Schachinger V, Schaefer HE (1994) Increased tissue endothelin immunoreactivity in atherosclerotic lesions associated with acute coronary syndromes. Lancet 344: 1405–1406. 7968078 10.1016/s0140-6736(94)90571-1

[30] Lerman A, Zeiher AM (2005) Endothelial function: cardiac events. Circulation 111: 363–368. 15668353 10.1161/01.CIR.0000153339.27064.14

[31] Ivey ME, Osman N, Little PJ (2008) Endothelin-1 signalling in vascular smooth muscle: pathways controlling cellular functions associated with atherosclerosis. Atherosclerosis 199: 237–247. 10.1016/j.atherosclerosis.2008.03.006 18436225

[32] Wu SY, Zhang BH, Pan CS, Jiang HF, Pang YZ, et al (2003) Endothelin-1 is a potent regulator in vivo in vascular calcification and in vitro in calcification of vascular smooth muscle cells. Peptides 24: 1149–1156. 14612185 10.1016/j.peptides.2003.07.008

[33] Peltonen T, Taskinen P, Napankangas J, Leskinen H, Ohtonen P, et al (2009) Increase in tissue endothelin-1 and ETA receptor levels in human aortic valve stenosis. Eur Heart J 30: 242–249. 10.1093/eurheartj/ehn482 19008257

[34] Li J, Flammer AJ, Nelson RE, Gulati R, Friedman PA, et al (2012) Normal vascular function as a prerequisite for the absence of coronary calcification in patients free of cardiovascular disease and diabetes. Circ J 76: 2705–2710. 22850339 10.1253/circj.cj-12-0683 PMC3786353

[35] Huang PH, Chen LC, Leu HB, Ding PY, Chen JW, et al (2005) Enhanced coronary calcification determined by electron beam CT is strongly related to endothelial dysfunction in patients with suspected coronary artery disease. Chest 128: 810–815. 16100172 10.1378/chest.128.2.810

[36] Essalihi R, Ouellette V, Dao HH, McKee MD, Moreau P (2004) Phenotypic modulation of vascular smooth muscle cells during medial arterial calcification: a role for endothelin? J Cardiovasc Pharmacol 44 Suppl 1: S147–150. 15838266 10.1097/01.fjc.0000166250.81733.a5

[37] Dao HH, Essalihi R, Graillon JF, Lariviere R, De Champlain J, et al (2002) Pharmacological prevention and regression of arterial remodeling in a rat model of isolated systolic hypertension. J Hypertens 20: 1597–1606. 12172322 10.1097/00004872-200208000-00023

[38] Cattaruzza M, Dimigen C, Ehrenreich H, Hecker M (2000) Stretch-induced endothelin B receptor-mediated apoptosis in vascular smooth muscle cells. FASEB J 14: 991–998. 10783154 10.1096/fasebj.14.7.991

[39] Moe SM, Reslerova M, Ketteler M, O'Neill K, Duan D, et al (2005) Role of calcification inhibitors in the pathogenesis of vascular calcification in chronic kidney disease (CKD). Kidney Int 67: 2295–2304. 15882271 10.1111/j.1523-1755.2005.00333.x

[40] Mustonen E, Pohjolainen V, Aro J, Pikkarainen S, Leskinen H, et al (2009) Upregulation of cardiac matrix Gla protein expression in response to hypertrophic stimuli. Blood Press 18: 286–293. 10.3109/08037050903244643 19919401

