# Supplementary material for: Association of Big Endothelin-1 with Coronary Artery Calcification
Source: PLoS One. 2015 Nov 13;10(11):e0142458. doi: 10.1371/journal.pone.0142458 (PMC4643989; doi:10.1371/journal.pone.0142458)
Supplement: S1 Table — (DOCX) [file pone.0142458.s001.docx]

**Supporting information**

**S1 Table** Comparison the level of CACS between ET-1 tertiles

| **Variable** | **CACS** | **p-value** |
| --- | --- | --- |
| Tertile 1 | 31.7±109.7 | <0.001 |
| Tertile 2 | 159.0±270.7 |  |
| Tertile 1 | 31.7±109.7 | <0.001 |
| Tertile 3 | 258.7±441.4 |  |
| Tertile 2 | 159.0±270.7 | <0.001 |
| Tertile 3 | 258.7±441.4 |  |

Bonferroni post hoc analysis was performed. Tertile 1: Patients with Plasma Big ET-1 level between 0.08 to 0.23 pmol/L; Tertile 2: Patients with Plasma Big ET-1 level between 0.24 to 0.52 pmol/L; Tertile 3: Patients with Plasma Big ET-1 level between 0.53 to 3.70 pmol/L.CACS: coronary artery calcium score
