# Supplementary material for: Association of Big Endothelin-1 with Coronary Artery Calcification
Source: PLoS One. 2015 Nov 13;10(11):e0142458. doi: 10.1371/journal.pone.0142458 (PMC4643989; doi:10.1371/journal.pone.0142458)
Supplement: S2 Table — (DOCX) [file pone.0142458.s002.docx]

**Supporting information**

**S2 Table** The10-year Framingham risk between groups

| **Variable** | **10-year Framingham risk** | **p-value** |
| --- | --- | --- |
| Group A | 6.9±6.2 | <0.001 |
| Group B | 10.5±8.0 |  |
| Group A | 6.9±6.2 | <0.001 |
| Group C | 13.0±11.7 |  |
| Group B | 10.5±8.0 | <0.001 |
| Group C | 13.0±11.7 |  |

Bonferroni post hoc analysis was used. Group A: CACS=0 and Tertile 1 of Big ET-1; Group C: CACS >0 and Tertile 3 of Big ET-1; Group B: Others. CACS: coronary artery calcium score
